# Increasing outdoor host-seeking in *Anopheles gambiae* over 6 years of vector control on Bioko Island

**DOI:** 10.1186/s12936-016-1286-6

**Published:** 2016-04-26

**Authors:** Jacob I. Meyers, Sharmila Pathikonda, Zachary R. Popkin-Hall, Matthew C. Medeiros, Godwin Fuseini, Abrahan Matias, Guillermo Garcia, Hans J. Overgaard, Vani Kulkarni, Vamsi P. Reddy, Christopher Schwabe, Jo Lines, Immo Kleinschmidt, Michel A. Slotman

**Affiliations:** Department of Entomology, Texas A&M University, College Station, TX USA; Medical Care Development International, Malabo, Equatorial Guinea; Department of Mathematical Sciences and Technology, Norwegian University of Life Sciences, Ås, Norway; Institut de Recherche Pour Le Développement (IRD), MIVEGEC, Montpellier, France; Department of Entomology, Faculty of Agriculture, Kasetsart University, Bangkok, Thailand; Medical Care Development International, Silver Spring, MD USA; MRC Tropical Epidemiology Group, London School of Hygiene and Tropical Medicine, London, UK; School of Pathology, Faculty of Health Sciences, University of Witwatersrand, Johannesburg, South Africa

**Keywords:** Outdoor host-seeking, *Anopheles gambiae*, *Anopheles coluzzii*, *Anopheles melas*, Bioko Island, Equatorial Guinea, Bioko Island Malaria Control Project, Indoor residual spraying, Vector control

## Abstract

**Background:**

Vector control through indoor residual spraying (IRS) has been employed on Bioko Island, Equatorial Guinea, under the Bioko Island Malaria Control Project (BIMCP) since 2004. This study analyses the change in mosquito abundance, species composition and outdoor host-seeking proportions from 2009 to 2014, after 11 years of vector control on Bioko Island.

**Methods:**

All-night indoor and outdoor human landing catches were performed monthly in the Bioko Island villages of Mongola, Arena Blanca, Biabia and Balboa from 2009 to 2014. Collected mosquitoes were morphologically identified and a subset of *Anopheles gambiae* sensu lato (*s.l.*) were later identified molecularly to their sibling species. Mosquito collection rates, species composition and indoor/outdoor host-seeking sites were analysed using generalized linear mixed models to assess changes in mosquito abundance and behaviour.

**Results:**

The overall mosquito collection rate declined in each of the four Bioko Island villages. *Anopheles coluzzii* and *Anopheles melas* comprised the *An. gambiae s.l.* mosquito vector population, with a range of species proportions across the four villages. The proportion of outdoor host-seeking *An. gambiae s.l.* mosquitoes increased significantly in all four villages with an average increase of 58.8 % [57.9, 59.64 %] in 2009 to 70.0 % [67.8, 72.0 %] in 2014. Outdoor host-seeking rates did not increase in the month after an IRS spray round compared to the month before, suggesting that insecticide repellency has little impact on host-seeking behaviour.

**Conclusion:**

While vector control on Bioko Island has succeeded in substantial reduction in overall vector biting rates, populations of *An. coluzzii* and *An. melas* persist. Host-seeking behaviour has changed in these *An. gambiae s.l.* populations, with a shift towards outdoor host-seeking. During this study period, the proportion of host-seeking *An. gambiae s.l.* caught outdoors observed on Bioko Island increased to high levels, exceeding 80 % in some locations. It is possible that there may be a genetic basis underlying this large shift in host-seeking behaviour, in which case outdoor feeding could pose a serious threat to current vector control programmes. Currently, the BIMCP is preparing for this potential challenge by testing source reduction as a complementary control effort that also targets outdoor transmission.

**Electronic supplementary material:**

The online version of this article (doi:10.1186/s12936-016-1286-6) contains supplementary material, which is available to authorized users.

## Background

The Bioko Island Malaria Control Project (BIMCP), funded by a consortium led by Marathon Oil Corporation and the Government of Equatorial Guinea, implemented a malaria control program on Bioko Island, Equatorial Guinea in 2004. The BIMCP employs vector control and malaria case management strategies to reduce and eventually eliminate malaria transmission on Bioko Island. Over the first 5 years of the malaria control programme there has been a 64 % reduction in malaria related death among under-5 year olds from 2004 to 2009 on Bioko Island [1].

At its inception, the BIMCP’s anti-vector intervention was based on indoor residual spraying (IRS) of pyrethroid insecticides. This resulted in the elimination of *Anopheles gambiae* S form and *Anopheles funestus* populations from the island [2]. While *Anopheles coluzzii* (formerly *An. gambiae* M form) and *Anopheles melas* remained on the island, their population sizes were drastically reduced [3, 4]. Though the BIMCP has been successful at reducing *Anopheles* populations and malaria incidence [1, 5], entomological inoculation rates (EIR) as high as 840 were observed on Bioko Island in 2009 [6]. However, the EIR has been reduced dramatically since then (unpublished results), even though malaria remains a major public health burden on the island.

The efficacy of IRS and LLIN interventions are predicated on the feeding and resting behaviour of the vectors [7]. *Anopheles coluzzii* and *An. melas* are both largely endophagic and endophilic [8]. However, a shift in mosquito host-seeking behaviours in response to anti-vector interventions has been observed in several malaria vectors in various parts of the world [9]. In several parts of its range, including India, Thailand, China and Vietnam, the Asian vector *Anopheles minimus* became more exophagic and exophilic, and in some cases more zoophilic and crepuscular, as a result of DDT spraying in the 1970s and 1980s [10]. Following these changes, anti-malarial spraying was reportedly less effective at interrupting transmission [7]. More recently, there have been several accounts of increased outdoor host-seeking in African *Anopheles* populations following an increase in vector control campaigns [10–15]. Some of these are probably phenotypic responses to the presence of excito-repellent insecticides, but some may be adaptive and due to evolved changes in genetically controlled behaviour—i.e., behavioural resistance (reviewed in [9]).

A limited study on Bioko Island in 1993 reported that outdoor (human landing catch) HLC collections caught no anophelines, while parallel indoor HLC collections caught between 3.7 and 6.0 anophelines (*An. gambiae s.l.* and *An. funestus*) per person/night [16]. However, these indoor/outdoor paired HLC collections were only conducted over two nights from three locations on Bioko Island [16]. Though the indoor/outdoor sampling was marginal, these observations suggested that mosquito feeding was occurring primarily indoors and that IRS and/or LLIN interventions would be highly effective [16, 17]. In 2009, the vector monitoring programme of the BIMCP was greatly expanded and paired indoor/outdoor HLC collections began in sentinel sites across the island. These HLC collections made clear that outdoor biting on Bioko Island was common in *An. coluzzii* and *An. melas* [6, 18].

The current study examines the effects of vector control on *An. coluzzii* and *An. melas* abundance and feeding behaviour on Bioko Island from 2009 to 2014. Entomological surveys were conducted and analysed across four locations in Bioko Island containing varying combinations of the two *Anopheles* vectors. Mosquito collections were analysed for changes in mosquito abundance, species composition and the proportion of outdoor host-seeking *An. gambiae s.l.* at paired indoor/outdoor HLC site. There was a decrease in the number of mosquitoes caught per collector night and an increase in the proportion host-seeking outdoors for both *An. coluzzii* and *An. melas* in all four villages. The hypothesis that this shift was a phenotypic response due to insecticide repellency was tested, and concluded that this is probably not the primary underlying cause of the observed changes. This leaves the possibility that the observed shift in feeding behaviour may be due to adaptive changes in the malaria vectors of Bioko Island.

## Methods

### Study area and indoor residual spraying programme

At its inception in 2004, the BIMCP’s anti-vector intervention was based on annual spraying of deltamethrin (Bayer Crop Science Inc., Isando South Africa) or alpha-cypermethrin (Fendona™, BASF South Africa PTY Ltd.) [2]. Following detection of the *kdr* allele in *An. gambiae* populations in 2005, along with an apparent lack of response to the IRS treatment by *An. gambiae,* the BIMCP decided to switch to bi-annual spraying of bendiocarb (Ficam™; Bayer Crop Science Inc), a carbamate insecticide, in 2009 (Additional file 1) [2]. Additionally, a single distribution round of long-lasting insecticidal nets (LLINs) was conducted in 2007, but coverage declined rapidly [1]. In 2013, BIMCP introduced a long-lasting encapsulated form of deltamethrin (K Orthrine SC 65, Bayer) to a subset of the Bioko Island villages (Additional file 1). This included the four villages in this study: Mongola (3°45′N, 8°43′E), Biabia (3°45′N, 8°42′E), Arena Blanca (3°30′N, 8°34′E) and Balboa (3°23′N 8°46′E). The Bioko Island map was created using the base maps in ArcGIS software (Esri software, Version 10.3).

The day before spray activities take a place, an advance team visit households in the targeted area, explain IRS, and inquire as to whether they would like their house sprayed the following day. Spray operators receive list of households that accepted to spray the following day. The IRS is completed with compression sprayers. Households that reject spraying or are absent on the day of spraying are revisited three times by the team in an attempt to get them to accept spraying.

### Mosquito collection

Human landing catches (HLCs) were performed from 2009 to 2014. In 2009, the HLCs were performed twice a month during March, May, July, September and November [18]. From 2010 onwards, HLCs were carried out monthly. In each village, mosquitoes were collected from 7 p.m. to 6 a.m. from four houses each separated by approximately 100 meters. It was only recorded whether each collection house was also sprayed by IRS in 2014 where each collection house received IRS.

Mosquitoes that approached the exposed lower leg of the HLC collector were mouth aspirated and stored for subsequent morphological identification. Each collection house had one HLC collector inside and another outside. At midnight, the indoor and outdoor collectors exchanged positions to control for collector bias. Two entomology field supervisors oversaw the collections to ensure that the volunteer collectors stayed awake during the night. Collectors were recruited from the communities of each village, were informed of the risks involved and were offered treatment if they showed symptoms of malaria. Consent was obtained from volunteer collectors verbally. Ethical approval for the vector monitoring was granted by authorities from the National Malaria Control Programme (NMCP) of the Equatorial Guinea Ministry of Health and Social Welfare.

### DNA extraction and diagnostics

Morphological identification was used to distinguish collected mosquitoes between Anophelines and Culicines. Anopheline mosquitoes were stored in 80 % ethanol and shipped to the laboratory for molecular analysis. Molecular identification was then used to distinguished sibling species identity within the *An. gambiae* complex. DNA extractions were performed with the QIAGEN tissue extraction kit on the QIAGEN Biosprint (QIAGEN Sciences Inc., Germantown, MD). Diagnostic PCR followed by a Hha*I* restriction enzyme digestion was used to identify the mosquito species of *An. gambiaes.l.* [19].

### Statistical models

#### Model 1: change in the number of host-seeking mosquitoes collected from paired indoor and outside sites from 2009 to 2014

Changes in the number of mosquitoes collected by HLC in indoor and outdoor sites from 2009 to 2014 were analysed by generalized linear mixed models (GLMMs) using the *glmmADMB* package in R [20–22]. The response variable of mosquitoes collected per collector-night was modelled by the fixed variables of year and site (indoor/outdoor). Month of collection was modelled as a random intercept to adjust for sampling variation of each month across years. Both mosquito per person night and year were treated as numerical variables. The fixed variable site was treated as a Bernoulli variable and the random variable, month, was treated as a factor. Overdispersion was tested using the *RVAideMemoire* package in R [20, 23]. The mosquito collection data was overdispersed as the residual deviance of 9200.724 was 74.8 times greater than the residual degrees of freedom of 123. The source of the overdispersion was associated with a wide range in the response value and not zero inflation as there were only fourteen observations of zero out of 616 observations. The data was fit to a negative binomial distribution to account for this overdispersion. Ninety-five percent credible intervals of the model coefficients were estimated from Markov chain Monte Carlo (MCMC) sampling of the posterior distribution of the model parameters with 50,000 MCMC simulations implemented in glmmADMB [20–22].

To compare the decrease in the number of host-seeking mosquitoes collected in each village over time, we created a compiled data set containing all of the villages and ran the above model with the additional fixed variables of village and the interaction of village and year. The models were tested with each village as the reference level for comparison. This approach was run on all mosquitoes from both indoor and outdoor collections individually and collectively. Village specific estimates were compared by the Wald test.

#### Model 2: change in the proportion of outdoor host-seeking mosquitoes from 2009 to 2014

Indoor/outdoor host-seeking behaviours and changes in species proportions were analysed by GLMMs assuming using the glmer function in the R package *lme4* [20, 24, 25]. We coded every individual mosquito collected as indoors or outdoors and modelled the probability of outdoor feeding using a binomial distribution by the fixed variable year. A random variable was conservatively designed that treated each paired indoor/outdoor collection at each collection house as an independent collection unit. The random variable individually coded every sampling event by time and location. This included collection year and month, collection village and house to best account for sampling bias caused by changes in housing condition, collector identity and meteorological conditions over the 6-year study period. Data on housing condition, collector identity and meteorological variables were not collected and could not be included in the model. The fixed variable, year, was treated as a numeric variable while site (indoor/outdoor host-seeking mosquitoes) was treated as a Bernoulli variable.

#### Model 3: change in the proportion of *Anopheles coluzzii* and *Anopheles melas* collected from 2009 to 2014

Since there were only two species of *An. gambiae s.l.* present, *An. coluzzii* and *An. melas*, the change in species composition was analysed using a GLMM assuming a binomial error distribution using the glmer function in the R package *lme4* [20, 24, 25]. This analysis was only performed on the *Anopheles* collected from Arena Blanca and Balboa because both Mongola and Biabia collections were almost exclusively *An. coluzzi*. The fixed and random variables, year and collection unit, utilized in this model were the same as described in Model 2. The response variable was the proportion of *An. coluzzii* mosquitoes out of all *An. gambiae s.l.* collected.

#### Model 4: change in the proportion of outdoor host-seeking mosquitoes in the month prior to an IRS spray event compared to the month following an IRS spray event from 2009 to 2014

To compare indoor/outdoor mosquito host-seeking behaviour in response to IRS events, the proportion of outdoor host-seeking mosquitoes in the month prior to an IRS spray round was compared to the month following an IRS spray round. The change in indoor/outdoor host-seeking behaviour was analysed by GLMM assuming a binomial error distribution using the glmer function in the R package *lme4* [20, 24, 25]. The fixed variable was the month (pre- or post-) of collection in relation to the IRS event. The response and random variables used in this model were the same as describe in Model 2.

For models 2–4, p values were estimated with a parametric bootstrap of the log-likelihood ratio of nested models with and without the effect of interest using the R package *pbkrtest*. All four models were run on each village separately and then on a compiled dataset to compare villages. For the compiled data set, the additional fixed variable of village was included into the GLMM as well as the interaction term between village and year. The most parsimonious model was selected by comparing the differential Akaike’s information criterion (dAIC < 2) and weight between the full model and all nested models.

## Results

### *Anopheles gambiae s.l.* collection rates decrease on Bioko Island from 2009 to 2014

**Fig. 1:**
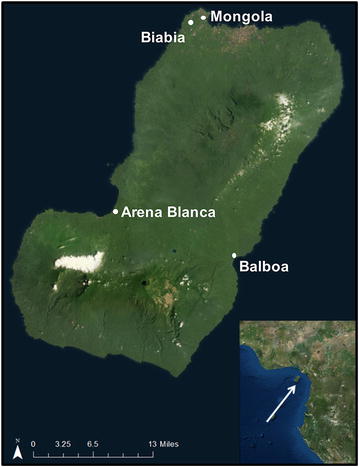
Map of Bioko Island. Mosquito surveillance was conducted in villages of Mongola, Arena Blanca, Biabia and Balboa on Bioko Island, Equatorial Guinea

A total of 34,874 *Anopheles gambiae s.l.* mosquitoes were collected by human landing catch from the villages of Mongola, Arena Blanca, Biabia and Balboa on Bioko Island between 2009 and 2014. Mongola and Biabia are located on the northwestern coast of the island, near the capital city of Malabo. Arena Blanca and Balboa are located on the south western and south eastern coasts, respectively (Fig. 1). Of the collected *Anopheles gambiae s.l*., 45.7 % were genotyped to species within the *An. gambiae* complex (Table 1). The mosquitoes selected for genotyping were equally distributed across the years and chosen at random within each month. Collections in Mongola and Biabia were predominantly *An. coluzzii* with almost no *An. melas* present (Table 1). Collections from Arena Blanca and Balboa each contained considerable numbers of both *An. coluzzii* and *An. melas*, though Arena Blanca contained predominantly *An. melas* while Balboa contained primarily *An. coluzzii* (Table 1). In addition, a very small number of *An. gambiaes.s.* (formerly S form) (n = 11 out of 15,951 mosquitoes analysed) were observed. These are suspected to have been migrants from the mainland.

**Table 1:** Mosquito collection numbers and species identification

	Mongola	Arena Blanca	Biabia	Balboa	Bioko Island
Total *An. gambiae s.l.* collected	10,970	9509	5024	9371	34,874
Number of *An. gambiae s.l.* molecularly identified to species	3091	4709	3039	5110	15,949
*An. coluzzii*	3068	862	3025	3698	10,653
*An. melas*	21	3842	13	1409	5285
*An. gambiae s.s.*	2	5	1	3	11

**Fig. 2:**
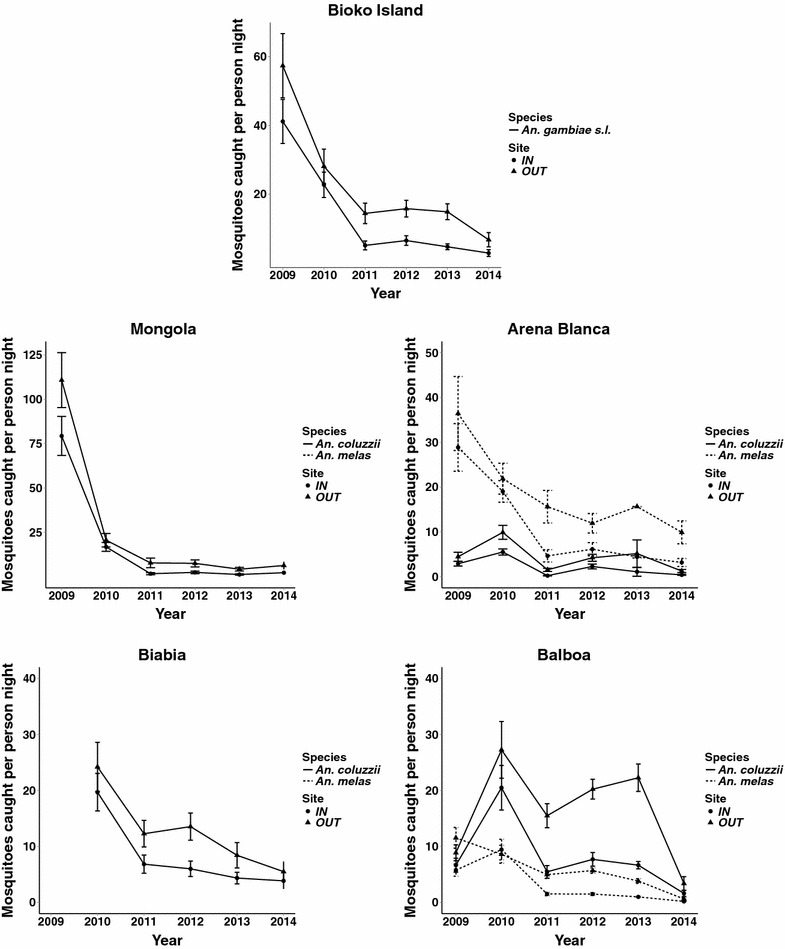
Indoor and outdoor *Anopheles* mosquito collection rates decline on Bioko Island from 2009 to 2014. HLC collection rate of *An. coluzzii* and *An. melas* from indoor and outdoor sites in Mongola, Arena Blanca, Biabia, Balboa and the average rate across the island. *Anopheles coluzzii* and *An. melas* species-specific collection rates are extrapolated from sub-sample of molecularly identified samples. Data presented as mean and standard error

The total number of *An. gambiae s.l.* collected on Bioko Island significantly declined from 2009 to 2014 (Table 2). The majority of this reduction occurred from 2009 to 2011 in both indoor (38.6 ± 5.4 to 5.5 ± 0.5 mosquitoes per person night) and outdoor collections (53.7 ± 7.4 to 15.7 ± 1.0 mosquitoes per person night) (mean ± standard error). Subsequently, *An.* gambiae *s.l.* collection rates remained relatively stable until 2014 in both indoor (2.8 ± 0.2 mosquitoes per person night) and outdoor collections (6.6 ± 0.6 mosquitoes per person night) (Fig. 2).

**Table 2:** Significant reduction in *An. gambiae s.l.* from indoor and outdoor collections in all four villages from 2009 to 2014

Site	Location	Abundance ratio^a^	95 % credible interval
Indoor	Mongola	0.4738	[0.3878, 0.5877]
	Arena Blanca	0.6402	[0.5418, 0.7451]
	Biabia	0.6462	[0.5571, 0.7753]
	Balboa	0.6226	[0.5353, 0.7168]
	Bioko Island	0.5760	[0.5222, 0.6415]
Outdoor	Mongola	0.5951	[0.5190, 0. 6948]
	Arena Blanca	0.7985	[0.6963, 0.8986]
	Biabia	0.7019	[0.6145, 0.8138]
	Balboa	0.7782	[0.6655, 0.8933]
	Bioko Island	0.7167	[0.6658, 0.7343]

^a^Abundance ratio, calculated as the exponentiated model coefficient and 95 % credible intervals, represents the relative annual change in *An. gambiae s.l.* abundance

*Anopheles gambiae s.l.* collection rates significantly decreased for both indoor and outdoor collections in each village individually (Fig. 2; Table 2). Mongola had the highest mosquito collection numbers of all villages in 2009 with 66.1 ± 13.9 mosquitoes per person night collected indoors and 92.3 ± 19.6 mosquitoes per person night collected outdoors. This was reduced to 2.2 ± 0.2 mosquitoes per person night collected indoors and 6.3 ± 0.6 mosquitoes per person night collected outdoors in 2014 (Fig. 2). A large proportion of this reduction, 77.2 % indoors and 83.4 % outdoors, occurred from 2009 to 2010.

Mosquito collections in Biabia, the neighbouring village to Mongola, only took place from 2010 to 2014. During that time, a similar decrease in *An. coluzzii* collection rates was in the two villages. Indoor collections declined from 19.7 ± 1.7 mosquitoes per person night in 2010 to 4.2 ± 0.4 mosquitoes per person night in 2014, while outdoor collections declined from 24.2 ± 1.9 mosquitoes per person night in 2010 to 6.0 ± 0.5 mosquitoes per person night in 2014 (Fig. 2).

Unlike Mongola and Biabia, which contained almost exclusively *An. coluzzii*, both *An. coluzzii* and *An. melas* represented a large proportion of the *An. gambiae s.l.* present in Arena Blanca and Balboa. The species proportions of *An. coluzzii* and *An. melas* among identified mosquitoes were extrapolated across the total number of *An. gambiae s.l.* collected in Arena Blanca and Balboa to estimate the species-specific effects of the BIMCP vector control programme on vector host-seeking. Since this data was extrapolated, it was not analysed by statistical models to justify significant changes and is limited to describe the observed changes in the extrapolated data. In Arena Blanca, there was roughly a four-fold reduction in both *An. melas* and *An. coluzzii* collection rates from 2009 to 2014 (Fig. 2). For *An. melas*, indoor collections diminished from 28.8 ± 6.7 mosquitoes per person night in 2009 to 3.4 ± 0.2 mosquitoes per person night in 2014, while outdoor collections were reduced from 36.4 ± 10.5 mosquitoes per person night to 10.8 ± 0.7 mosquitoes per person night. For *An. coluzzii*, indoor collections diminished from 2.8 ± 0.7 mosquitoes per person night in 2009 to 0.4 ± 0.1 mosquitoes per person night in 2014, while outdoor collections were reduced from 4.4 ± 1.3 mosquitoes per person night to 1.4 ± 0.1 mosquitoes per person night (Fig. 2).

In Balboa, *An. coluzzii* collection rates increased in both indoor (6.6 ± 1.6 to 20.5 ± 0.7 mosquitoes per person night) and outdoor collection (8.9 ± 1.8 to 27.2 ± 1.0 mosquitoes per person night) from 2009 to 2010. Both collections stayed relatively high until 2014 where indoor collections dropped to 1.8 ± 0.2 mosquitoes per person night for indoor collections and 3.7 ± 0.4 mosquitoes per person night for outdoor collections. *An. melas* collection rates did not increase during this same time period in Balboa as indoor collections decreased from 5.8 ± 1.4 to 0.2 ± 0.02 mosquitoes per person night and outdoor collections decreased from 11.5 ± 2.3 to 0.7 ± 0.1 mosquitoes per person night (Fig. 2).

In comparing the decrease in *An. gambiae s.l.* collected per person night between villages, there was a significantly greater reduction in Mongola compared to the other three villages (Table 3). This translated to both indoor and outdoor collections, except for outdoor collection in Biabia, where the decrease in mosquitoes collected per person night was only slightly distinct from Mongola (p value = 0.0711) (Table 3).

**Table 3:** The decrease in *An. gambiae s.l.* collected per person night is significantly higher in Mongola compared to Arena Blanca, Biabia and Balboa

Site	Village compared to Mongola	Z-score	p values
All HLC	Arena Blanca	4.48	<0.0001
	Biabia	2.77	0.0056
	Balboa	4.02	<0.0001
Indoor HLC	Arena Blanca	3.34	0.0008
	Biabia	3.32	0.0009
	Balboa	3.13	0.0018
Outdoor HLC	Arena Blanca	3.71	0.0002
	Biabia	1.81	0.0711
	Balboa	3.27	0.0011

### The proportion of *Anopheles gambiae s.l.* caught outdoors in paired indoor-outdoor catches increased on Bioko Island 2009–2014

**Fig. 3:**
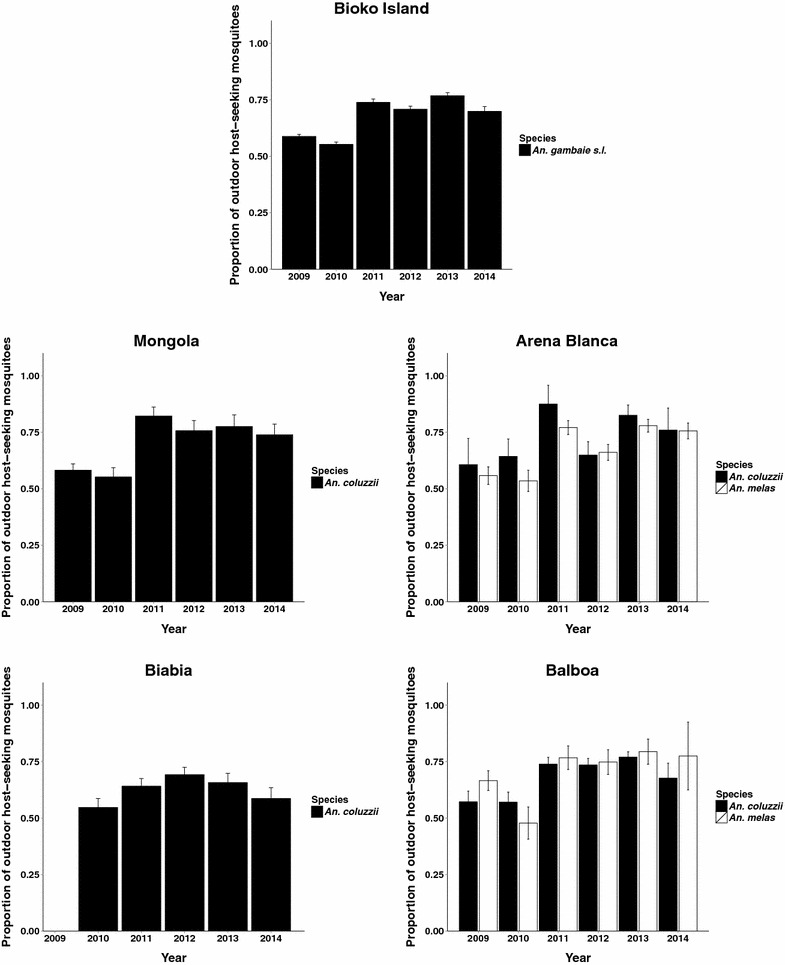
Increase in outdoor host-seeking proportions on Bioko Island from 2009 to 2014. The proportion of outdoor host-seeking *An. coluzzii* and *An. melas* in Mongola, Arena Blanca, Biabia, Balboa and compiled across Bioko Island. Data presented as mean and 95 % confidence intervals

While the number of both indoor and outdoor feeding *An. gambiae s.l.* decreased over time, the proportion of *An. gambiae* s*.l.* collected outdoors increased significantly (Fig. 3; Table 4). The proportion of outdoor host-seeking mosquitoes was computed as the number of mosquito collected from outdoor HLC divided by the overall number of mosquitoes collected by both outdoor and indoor HLC. Change in the probability of *An. gambiae s.l.* host-seeking indoors *versus* outdoors was analysed by year using generalized linear mixed models assuming a binomial error distribution. Overall, there was an increase in the proportion of outdoor-collected mosquitoes on Bioko Island from 58.8 % [57.9, 59.64 %] in 2009 to 70.0 % [67.8, 72.0 %] in 2014 (p value = 0.00052). On average, the odds of being captured outdoors rather than indoors increased in *An. gambiae s.l.* by a factor of 1.19 annually from 2009 to 2014 (Table 4).

**Table 4:** Odds ratio of outdoor host-seeking relative to indoor host-seeking, by village from 2009 to 2014

	Odds ratio	95 % confidence interval	p values
Mongola	1.27	[1.19, 1.36]	0.0005147
Arena Blanca	1.28	[1.20, 1.37]	0.0005420
Biabia	1.08	[1.02, 1.16]	0.02662
Balboa	1.21	[1.15, 1.28]	0.0005277
Bioko Island	1.19	[1.15, 1.23]	0.0005192

The proportion of *An. gambiae s.l.* captured outdoors also increased significantly in each village individually (Table 4). Mongola, Arena Blanca and Balboa had very similar increases in the proportion of outdoor host-seeking *An. gambiae s.l.* from 2009 to 2014. While Biabia had a significant increase in outdoor host-seeking mosquitoes (odds ratio: 1.08 [1.02, 1.16], it was distinctly lower than Mongola (odds ratio: 1.27 [1.19, 1.36]) and Arena Blanca (odds ratio: 1.28 [1.20, 1.37]) (Table 4).

**Fig. 4:**
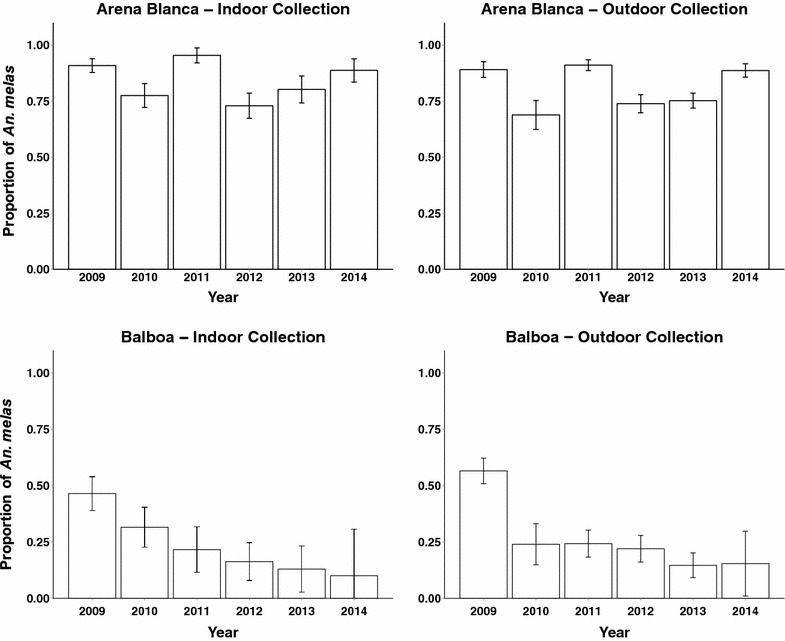
Relative proportion of *Anopheles melas* to *Anopheles coluzzii* is reduced in Balboa, but not Arena Blanca, from indoor and outdoor sites from 2009 to 2014. The relative proportion of *An. melas to An. coluzzii* in indoor and outdoor collections from Arena Blanca and Balboa from 2009 to 2014. Data presented as mean and 95 % confidence intervals

Significant changes in the species proportions of *An. coluzzii* and *An. melas* within indoor and outdoor collections could indicate species-specific behavioural changes in host-seeking from 2009 to 2014. To examine this, the proportion of *An. coluzzii* to *An. melas* in indoor and outdoor collections from 2009 to 2014 were compared using GLMMs assuming a binomial distribution (Fig. 4). This analysis was only performed on Arena Blanca and Balboa because the Mongola and Biabia collections contained a negligible number of *An. melas* (Table 1). In Arena Blanca, the proportion of *An. melas* to *An. coluzzii* in the indoor (odds ratio: 0.95 [0.88, 1.03], p value = 0.160) and outdoor collections did not change (odds ratio: 0.96 [0.91, 1.02], p value = 0.188) (Fig. 4). This indicates that there were no species-specific changes in outdoor host-seeking behaviour in Arena Blanca. In Balboa, the proportion of *An. melas* in both indoor (odds ratio: 0.66 [0.61, 0.71], p value < 0.001) and outdoor collections (odds ratio: 0.65 [0.62, 0.69], p < 0.001) decreased significantly (Fig. 4). Since the proportion of *An. melas* decreased in a similar manner in both indoor and outdoor collections, this indicates an overall reduction in *An. melas* in this population over time, rather than a species-specific behavioural shift in host-seeking behaviour.

**Fig. 5:**
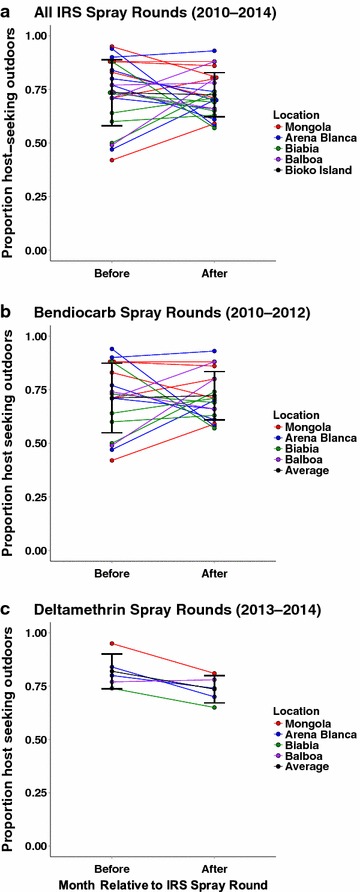
IRS does not have a direct effect on outdoor host-seeking proportions in months surrounding IRS spray rounds. **a** Outdoor host-seeking proportions in the month before an IRS spray event and the month after an IRS spray event from 2010 to 2014. Data separated to compare outdoor host-seeking proportions while **b** bendiocarb or **c** deltamethrin insecticides were utilized as the IRS insecticide

It is possible that the observed outdoor host-seeking behaviour might be caused by repellency of the insecticide used in IRS. To examine this, the probability of *An. gambiae s.l.* outdoor host-seeking in the month before was compared to the month following IRS spray rounds, using GLMMs assuming a binomial error distribution (Fig. 5a). The expectation is that if insecticide repellency has a noticeable impact on host-seeking behaviour, there would be a higher proportion of outdoor host-seeking immediately following a spray round. Overall, there was no significant difference between the proportion of host-seeking mosquitoes caught outdoors in the month before IRS (0.73 ± 0.15 %) to that in the month following an IRS (0.72 ± 0.10 %) (odds ratio: 0.92 [0.76, 1.11], p value = 0.376) (Fig. 5a).

In 2013, the BIMCP re-introduced an encapsulated, long-lasting formulation of deltamethrin as the active insecticide for IRS, replacing bendiocarb. In order to examine the specific repellency effects of each insecticide, separate analyses were performed for 2010–2012 when bendiocarb was used, and for 2013–2014 when deltamethrin was used. The models suggest that IRS treatments with bendiocarb had no apparent effect on the proportion of host-seeking *An. gambiae s.l.* caught outdoors after spraying (odds ratio: 0.99 [0.80, 1.24], p value = 0.985) (Fig. 5b). Surprisingly, when deltamethrin was utilized in IRS in 2013 and 2014, the proportion of host-seeking mosquitoes caught outdoors declined significantly in the month following spraying (odds ratio: 0.71 [0.52, 0.97], p value = 0.0338) (Fig. 5c).

## Discussion

Significant changes in *An. gambiae s.l.* vector abundance and host-seeking behaviour occurred under the BIMCP between 2009 and 2014. During this time, the number of *An. gambiae s.l.* collected per person night was reduced by 93 and 87 %, for indoor and outdoor collections respectively. The BIMCP began in 2004 and has reduced mosquito populations dramatically over its first 5 years [2–4]. This data indicates that the BICMP has continued to reduce malaria mosquito populations, 6 years into the project. By 2014, all four villages had, on average, under ten *An. gambiae s.l.* collected per person night. Although current vector control tools used on Bioko are only applied indoors, there was a decrease in the rate of both indoor and outdoor host-seeking mosquitoes. In comparing the decrease in mosquitos collected per person night between villages, Mongola had a larger reduction in host-seeking mosquitoes compared to Arena Blanca, Biabia and Balboa. This appears to be due to the large number of host-seeking *An. gambiae s.l.* present at the beginning of the study in Mongola compared to the other villages (Fig. 2).

During this time, the proportion of host-seeking *An. gambiae s.l.* caught outdoors, in paired indoor-outdoor human landing catches, has increased. On Bioko Island, the proportion caught outdoors in paired HLC catches has now reached over 80 % in some locations: this is higher than other recent reports of outdoor feeding proportions in response to IRS or LLINs [10–15]. For example, in Benin, following large scale LLINs distribution, the proportion of *An. funestus* caught outdoors in paired human-landing catches increased from 45 % in 2008 to 68.1 % in 2011 [13]. In Tanzania, following two LLINs distributions, this proportion increased from 39.2 % in 1997 to 70.2 % in 2009 in *An. funestus*, although *An. gambiae* host-seeking behaviour did not change during this same period [10].

High outdoor biting rates were reported for *An. coluzzii* (then referred to as *An. gambiae* M) on Bioko Island in 2009 [6, 18]. This contrasted with reports from Bioko prior to 2004, in which outdoor biting was not observed [16, 17]. This implies a behavioural shift favoring outdoor host-seeking, possibly as the result of intense selection pressure imposed by the indoor application of insecticides. This observation was consistent with other studies both in Africa and elsewhere reporting an increased proportion of outdoor host-seeking in response to IRS or LLINs [10–15]. Here, we have shown that a shift in host-seeking behaviour has accompanied the IRS-based vector control programme under the BIMCP. Assuming that there was no systematic change over the study period in the methods used for human landing catches (including collector bias), or in the relative accessibility of the collectors stationed indoors and outdoors, this change in the proportion caught outdoors implies a change in the intrinsic behaviour of the local mosquitoes.

If this kind of behavioural shift is inherited, adapted and evolved, then it is worrisome. However, it is important to emphasize that so far, the substantial overall reduction in *Anopheles* mosquito populations in Bioko has resulted in a decrease in the total number of outdoor blood meals (Fig. 2) [26]. A recent analysis, that combined human night-time behaviour with mosquito host-seeking behaviour, showed that malaria transmission on Bioko Island is still primarily driven by indoor contact between vectors and humans [26]. This is because humans are primarily indoors during the times when mosquitoes are searching for hosts. This is consistent with the malaria transmission studies conducted in Burkina Faso, Tanzania, Kenya, and Zambia (including those mentioned above) where almost all human exposure to *An. gambiae* complex mosquitoes and *An. funestus* occurred indoors, even though outdoor biting rates were high during collections [27].

Nonetheless, although outdoor biting is not currently the primary site of malaria transmission, it is recognized as a considerable potential problem for the future. It is now widely accepted that outdoor transmission will need to be addressed in order to achieve the goal of eliminating malaria from many endemic areas [9, 28, 29]. Indeed, it has been reported that among people protected by LLINs at night, about half of the exposure to malaria vectors occurs outdoors [30, 31]. Furthermore, simulation studies have suggested that increased outdoor biting, if it is inherited and thus a form of behavioural resistance, may have as large an impact on our ability to control vectors as physiological forms of insecticide resistance [32].

In some situations, a plausible explanation for an increase in outdoor host-seeking of malaria vectors during vector control programmes is the reduced availability of host indoors, e.g., because they are protected by bed nets. However, vector control on Bioko Island has consisted primarily of IRS, with average bed net coverage being low [1]. Unless mosquitoes are greatly deterred or repelled by the insecticide, IRS does not make hosts unavailable to host-seeking mosquitoes. If the IRS insecticide exhibits deterrency/repellency effects on host-seeking *An. gambiae s.l.,* there would presumably be an increase in outdoor host-seeking in the month following an IRS spray round relative to the month prior, assuming that the houses used for sampling were sprayed. However, when the proportion of host-seeking mosquitoes caught outdoors in the months surrounding an IRS spray round was analysed, there was no increase in this proportion following spraying, regardless of the insecticide sprayed. Indeed, there was an increase in the proportion of indoor host-seeking mosquitoes in the month following a deltamethrin spray compared to the month prior. It is unlikely that this change in host-seeking behaviour is due to declining insecticide residues in the homes as previous reports suggest that IRS spray of bendiocarb remain on the interior walls of homes for 3 months and the long-lasting encapsulated deltamethrin is expected to last even longer [33]. From 2009 to 2013, the houses which were sprayed were not individually recorded. This could undermine the power of this analyses. In 2014, the status of collection houses was recorded and all houses used in the collections that year were indeed sprayed. A large number of collections were included in this analyses (92 houses) and undoubtedly the vast majority of them would have been covered during the spray rounds. Therefore, it is unlikely there were a large number of unsprayed houses included in the collections which might confound the analysis. Currently, there is no clear explanation for why the proportion of indoor host-seeking mosquitoes would increase in the month following a deltamethrin spray compared to the month prior and future work will be required to uncover the basis for this behaviour.

If the behaviour of outdoor-host-seeking is heritable, and if outdoor hosts are available, then presumably the suppression of mosquito populations by heavy usage of IRS or LLINs could select for a mainly outdoor-feeding *Anopheles* population. If such a population emerged, it would be fully adapted to avoid indoor-applied insecticide. This could result in a resurgence of malaria transmission and the failure of programmes based on current vector control tools [9]. It cannot be assumed that the increased outdoor feeding has an underlying genetic basis, but this possibility is clearly of great concern.

## Conclusions

It is clear that in years six through eleven of the BIMCP vector control programme, *Anopheles gambiae s.l.* populations have significantly decreased on Bioko Island. At the same time, the proportion of this reduced population caught outdoors has increased to high levels. This data suggests that this shift is probably not attributable to repellency by the insecticide used for IRS. At the moment, vector control remains effective despite this increased outdoor feeding behaviour [26]. Nevertheless, if it has a genetic basis, then it must be regarded as a critical cause of concern for the future. With the evidence reported here, there is a real possibility that this behavioural shift represents an adaptive evolved response. Ongoing work will likely provide the answer to whether outdoor host-seeking behaviour is due to an evolutionary adaptation to vector control. If true, outdoor host-seeking could become a threat to the progress made by the BIMCP in reducing malaria transmission and could lead to a rebound in infection in the absence of an effective outdoor control strategy. Regardless, control measures that target outdoor feeding may be needed to eventually eliminate residual transmission on Bioko Island. To counter a future threat of outdoor feeding mosquitoes and to continue progress in the reduction of malaria transmission, the BIMCP has recently added a pilot initiative in larval control to its vector control strategy.

## Additional file

10.1186/s12936-016-1286-6 Month and year of IRS spray rounds by village.
