# Supplementary material for: Increasing outdoor host-seeking in Anopheles gambiae over 6 years of vector control on Bioko Island
Source: Malar J. 2016 Apr 26;15:239. doi: 10.1186/s12936-016-1286-6 (PMC4845310; doi:10.1186/s12936-016-1286-6)
Supplement: Supplementary file 1 — 10.1186/s12936-016-1286-6 Month and year of IRS spray rounds by village. [file 12936_2016_1286_MOESM1_ESM.docx]

**Additional file 1. Month and year of IRS spray rounds by village.**

|  | 2009 | 2010 | 2011 | 2012 | 2013 | 2014 |
| --- | --- | --- | --- | --- | --- | --- |
| Mongola | Oct. | Mar. | Jun., Nov. | Apr., Sep. | Jun. | Mar. |
| Arena Blanca | Nov. | Feb. | May, Nov. | Apr., Sept. | Apr. | Jun. |
| Biabia | Oct. | Dec. | Apr., Sep. | Apr., Sep | Jun. | Mar. |
| Balboa | Oct. | Dec. | May, Oct |  | Jul. | Mar. |

Bendiocarb was utilized as the IRS insecticide from 2009-2012. Encapsulated, long-lasting deltamethrin was utilized in 2013-2014.
